# The Influence of Tranexamic Acid (TXA) on Postoperative Infection Rates Following Total Hip Arthroplasty (THA)—A Systematic Review

**DOI:** 10.3390/jcm14092910

**Published:** 2025-04-23

**Authors:** Radu Prejbeanu, Mihail Lazar Mioc, Eleftherios Tsiridis, Eustathios Kenanidis, Federico Valli, Andrea Pasquini, Bogdan Deleanu

**Affiliations:** 1 Department XV Orthopedics and Traumatology, “Victor Babes” University of Medicine and Pharmacy, Eftimie Murgu Square, No. 2, 300041 Timisoara, Romania; 2 Academic Orthopaedic Department, Aristotle University Medical School, General Hospital Papageorgiou, Ring Road Efkarpia, 56403 Thessaloniki, Greece; 3 IRCCS Istituto Ortopedico Galeazzi, Via Cristina Belgioioso, 173, 20157 Milano, MI, Italy; 4 Center for Modeling Biological Systems and Data Analysis, “Victor Babes” University of Medicine and Pharmacy, Eftimie Murgu Square, No. 2, 300041 Timisoara, Romania; 5 Department of Orthopaedics and Traumatology, “Pius Brinzeu” Emergency Clinical County Hospital, Bld Liviu Rebreanu, No. 156, 300723 Timisoara, Romania

**Keywords:** tranexamic acid, TXA, total hip arthroplasty, THA, surgical site infection, SSI periprosthetic joint infection, PJI, postoperative complications, postoperative infection

## Abstract

**Background:** Tranexamic acid (TXA) has become a cornerstone in total hip arthroplasty for reducing blood loss and minimizing transfusion requirements. However, its influence on postoperative infection rates, including surgical site infections and periprosthetic joint infections (PJIs), remains a topic of debate. This systematic review aims to explore the association between tranexamic acid use and infection rates in total hip arthroplasty. **Methods:** Following PRISMA 2020 guidelines, an electronic search was performed in the PubMed, Scopus, Web of Science, Cochrane, and Epistemonikos databases. A PICO-based question was developed to select relevant studies, which were assessed for quality using the MINORS tool for non-randomized studies and the RoB 2 tool for randomized controlled trials (RCTs). This review critically appraises three studies, including one RCT and two retrospective cohort studies. **Results:** Of 277 studies identified, 3 met inclusion criteria, totaling 146,227 patients. Findings indicate that tranexamic acid is generally associated with reduced periprosthetic joint infections and surgical site infection rates, with some variability based on administration routes and dosages. Despite these promising results, methodological limitations in the included studies underscore the need for further high-quality research to establish optimal tranexamic acid protocols. **Conclusions:** In summary, this systematic review indicates that TXA could reduce postoperative infection rates following total hip arthroplasty (THA). Further well-designed randomized controlled trials are required to validate these findings and determine the best dosing and administration strategies. PROSPERO registration: CRD42024589078.

## 1. Introduction

Tranexamic acid (TXA) has become an essential pharmacological agent in hip arthroplasty. It is credited for its efficacy in reducing perioperative blood loss and the subsequent need for transfusions [1,2,3,4]. Tranexamic acid (TXA) is a synthetic lysine analog antifibrinolytic that inhibits plasminogen activation, reducing fibrinolysis and promoting clot stability. Its role in preserving hemostasis during and after orthopedic procedures has been well-supported by recent research, particularly in total joint arthroplasty, where it has been shown to minimize perioperative blood loss and reduce transfusion requirements [5,6]. Despite this, the potential for TXA to influence postoperative infection rates remains a controversial issue within the medical community. Surgical site infections (SSIs) are a significant concern, as they can lead to adverse patient outcomes, including prolonged hospital stays, additional surgeries, and increased healthcare costs [7,8,9]. Moreover, the association between SSIs and long-term health outcomes such as hospital readmissions and mortality, along with the healthcare costs attributed to SSIs even one year following surgery, underlines the gravity of these postoperative complications [10].

The debate centers on whether TXA’s blood-conserving effects translate into a lower incidence of infections. This may be due to a variety of interconnected factors, such as reduced exposure to allogeneic blood transfusions and their immunomodulatory effects. Moreover, there is a need to examine the influence of different administration routes, dosages, and timing of TXA on infection rates, as current evidence suggests no single approach is superior in blood-sparing properties.

This systematic review aimed to evaluate the current evidence regarding the effect of TXA on postoperative infection rates following hip arthroplasty. By critically assessing the quality and findings of the available literature, this analysis aims to provide insights into TXA’s role in reducing infection risk and informing evidence-based clinical practices in orthopedic surgery. We hypothesize that the use of TXA in THA will decrease overall postoperative infection rates.

## 2. Materials and Methods

To ensure a focused and effective search strategy, we structured our research question using the PICO framework, which defines the following:

- P—Population: Adult patients requiring primary total hip arthroplasty.
- I—Intervention: Administration of TXA prior or at the time of surgery, no matter the route of administration.
- C—Comparison: Patients undergoing primary total hip arthroplasty without the administration of TXA would serve as the control group.
- O—Outcome: The rate of postoperative infections which can be defined as postoperative infection, surgical site infection, or periprosthetic joint infection.

This framework guided the development of our inclusion and exclusion criteria and informed the construction of our search strategy, ultimately facilitating a comprehensive and systematic approach to literature retrieval and analysis.

A comprehensive search was conducted from inception until 25 December 2024 through PubMed, Web of Science, Scopus, Cochrane, and Epistemonikos databases to identify relevant studies evaluating the impact of TXA on postoperative infection rates following total hip arthroplasty (THA). The search strategy was meticulously designed to include a combination of keywords and MeSH terms such as “tranexamic acid”, “total hip arthroplasty”, and “postooperative infection”. See Supplementary File S1 for the extended form. We registered our study in the PROSPERO database with the CRD42024589078 number.

Screening and consensus on eligibility were carried out by two authors (MLM and AP). Any dispute was resolved by discussion between these two authors. See Supplementary File S2 for the extended form.

In the systematic review, several exclusion criteria were applied to ensure that only the most relevant studies were included for analysis. Studies were excluded if they were categorized as literature reviews, systematic reviews, or meta-analyses, as these types of research synthesize existing data rather than present original findings. Articles that lacked an abstract and could not be sufficiently evaluated for inclusion based on title alone were excluded. This step was taken to ensure consistency and reproducibility in the screening process across all identified records. Editorials, comments, non-human studies, and pediatric studies were also excluded, as they do not pertain to adult human subjects undergoing THA for infection rate assessment following TXA administration. Furthermore, conference abstracts were omitted due to their typically limited methodological details and lack of peer review.

Additionally, specific surgical scenarios such as revision arthroplasty, joint replacement for hip trauma, or any knee arthroplasty studies were excluded, as these may involve different patient populations, surgical procedures, or outcomes that are not directly comparable to primary THA. Studies that involved additional procedures alongside primary THA were also excluded to avoid confounding factors that could influence infection rates. Research focusing on associated conditions such as tumors or coagulation disorders was excluded to maintain focus on studies evaluating infection outcomes in otherwise healthy individuals undergoing elective THA. Moreover, studies involving bibliometric analysis or break-even analysis were excluded, as these are economic or publication trend assessments rather than clinical outcome studies. Finally, studies were excluded if they involved the wrong intervention (i.e., no use of TXA) or if they measured outcomes other than infection rates (the primary endpoint of interest) Table 1.

We aimed to analyze each component of our manuscripts’ results. Where possible, we also performed an analysis based on TXA dosage and route of administration.

**Figure 1:**
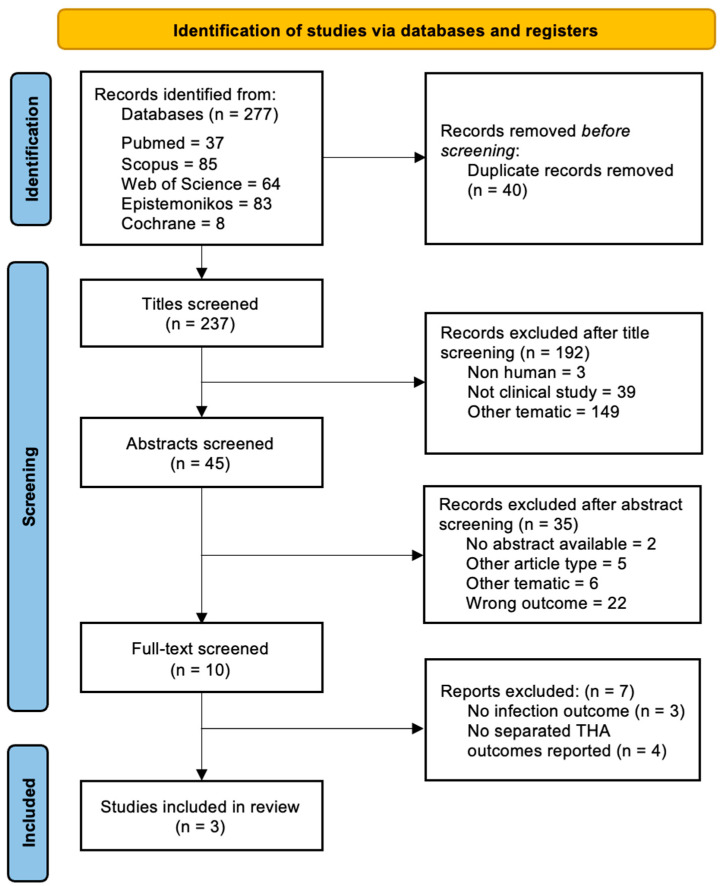
PRISMA flowchart, depicting the identification and screening of our studies.

We adhered to the PRISMA (Preferred Reporting Items for Systematic Reviews and Meta-Analyses) guidelines [11]. This adherence was reflected in our meticulous documentation of the search strategy, selection criteria, screening process, and methods of resolving discrepancies. The PRISMA guidelines provided a framework for ensuring that our review was conducted with the utmost methodological rigor and transparency, facilitating reproducibility and trust in our literature synthesis. The entire identification and screening process can be seen in our PRISMA flowchart (Figure 1). Two reviewers (FV and DB) independently assessed the quality of each included study. Risk of bias and manuscript quality were evaluated with MINORS—for retrospective cohort studies and RoB2—for RCTs [12,13]. The RoB2 assessment was visually presented with the aid of the Robvis tool [14].

## 3. Results

Three studies were included in the final analysis [15,16,17], with varying levels of evidence, ranging from retrospective cohort studies to randomized controlled trials (RCTs), as can be seen in the PRISMA flowchart (Figure 1).

These studies primarily investigated the effects of TXA administration in the context of THA, focusing on outcomes such as surgical site infections (SSIs) and PJI, alongside the dosage and route of administration of TXA. The baseline characteristics of the included studies have been synthesized in Table 2, while the results have been synthesized in Table 3.

### 3.1. Dosage and Route of Administration

The included studies assessed various TXA administration routes and dosages, reflecting the heterogeneity in clinical practice. Hsu et al. evaluated IV TXA, administering 1 g IV at induction followed by a second 1 g dose three hours later, as well as topical TXA at a single intraoperative dose of 2 g. Thapaliya et al. also focused on IV TXA, using a single 1 g dose intraoperatively, while their topical TXA protocol involved 3 g applied directly to the surgical site before closure. Unfortunately, they did not specify the number of patients in each of these subgroups. Luo et al. was the only study to examine oral TXA, administering 2 g orally two hours preoperatively, followed by two postoperative doses of 1 g each. This study compared oral TXA to topical TXA, where a single dose of 3 g was applied intraoperatively.

### 3.2. Surgical Site Infections

One study reported on the impact of TXA on SSI rates:

Thapaliya et al. indicated that at 30 days, the incidence of superficial SSI was 0.1% in the TXA group, significantly lower than in the non-TXA group (*p* < 0.001). By 90 days, the superficial SSI rate in the TXA cohort increased to 0.3% (*p* < 0.001). For deep SSI, no significant difference was found at 30 days (0.1%, *p* = 0.228), but at 90 days, the TXA group had a lower deep SSI rate of 0.1% (*p* = 0.012).

### 3.3. Periprosthetic Joint Infections

Three studies assessed the effect of TXA on PJI rates:

Hsu et al. conducted a retrospective analysis to evaluate the impact of TXA on PJI rates specifically in THA patients. In this subgroup, TXA administration was associated with a significantly lower incidence of PJI compared to the non-TXA group (TXA: 0.9%, non-TXA: 1.8%, *p* = 0.004). After adjusting for confounders, multivariate analysis confirmed that TXA use was linked to a 50% reduction in PJI risk (OR 0.50, 95% CI: 0.28–0.88, *p* = 0.017). Both intravenous and topical TXA independently contributed to this protective effect.

Thapaliya et al. found that at 30 days postoperatively, the TXA group exhibited a significantly lower risk of PJI compared to the non-TXA group (RR 0.808, 95% CI 0.710–0.920, *p* = 0.001). This protective effect persisted at 90 days, with TXA use associated with a continued reduction in PJI risk (RR 0.894, 95% CI 0.815–0.982, *p* = 0.019).

In a 117-patient cohort, Luo et al. reported no cases of PJI in either the oral or topical TXA groups during the 90-day follow-up period. These findings suggest that TXA, regardless of the administration route, does not appear to increase the risk of PJI. Additionally, both oral and topical TXA were found to be equivalent in reducing perioperative blood loss, without any significant differences in transfusion rates or thromboembolic complications.

### 3.4. Risk of Bias and Quality Assessment

For the two non-randomized studies, MINORS scores ranged from 10 to 14, indicating moderate methodological quality (Table 4). Hsu et al. had limitations, including the lack of consecutive patient inclusion and the absence of unbiased outcome assessment, which may introduce selection and measurement bias. However, it prospectively collected data, had appropriate study endpoints, and ensured complete follow-up. Thapaliya et al., with a higher MINORS score of 12, demonstrated better methodological rigor, including consecutive patient inclusion, unbiased outcome assessment, and complete follow-up, but lacked prospective data collection, which could impact study reliability.

**Figure 2:**
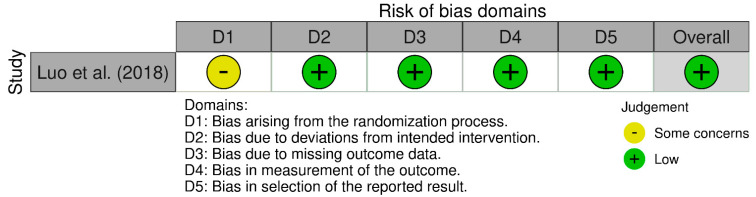
RoB 2 assessment visualized with the Robvis tool for our RCTs [17].

For the single randomized controlled trial, Luo et al., the RoB 2 assessment showed an overall low risk of bias, with some concerns in the randomization process due to unclear allocation concealment. However, other bias domains, including deviations from the intended intervention, missing outcome data, measurement of outcomes, and selection of reported results, were all rated as low risk, indicating a well-controlled and methodologically sound trial (Figure 2).

Overall, the methodological quality of the included studies varied, necessitating cautious interpretation of the findings. The RCT showed strong methodological rigor with a low risk of bias, though some concerns regarding the randomization process remain. In contrast, the non-randomized studies exhibited moderate quality. MINORS scores ranged from 10 to 14, indicating potential bias risks due to retrospective designs, lack of prospective data collection, and possible selection biases.

## 4. Discussion

The findings from this systematic review highlight a potential role of TXA in reducing infection rates following THA, with all three included studies demonstrating a favorable association between TXA use and lower PJI rates. The cohort studies reported a significant reduction in PJI risk with intravenous and topical TXA administration, while the randomized controlled trial (RCT) found no PJI cases in either oral or topical TXA groups, reinforcing its safety regarding infection risk in THA. However, variability in surgical site infection (SSI) outcomes suggests that TXA’s role in superficial infections requires further investigation. Thapaliya et al. observed lower superficial SSI rates at 30 days in TXA recipients, but by 90 days, superficial SSI rates were slightly elevated, raising concerns about long-term wound healing and potential delayed complications associated with TXA use. The possibility that TXA may impair fibrinolysis at the surgical site warrants further research, particularly regarding its effects on superficial wound healing dynamics [18].

**Figure 3:**
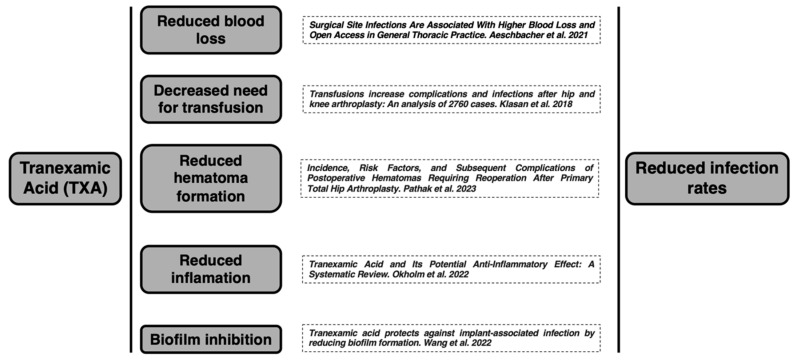
Graphical representation of TXA pathways that reduce postoperative infection rates [19,20,21,22,23].

The complexity of postoperative infection etiology requires a rigorous investigation, unencumbered by the confounding variables present in clinical settings (Figure 3). In vitro studies provide a controlled environment to investigate specific biological mechanisms, such as the effect of tranexamic acid on fibrinolysis, coagulation, bacterial proliferation, or biofilm formation, independent of the many confounding factors present in clinical settings. By replicating the conditions of TXA use and surgical intervention without these confounders, in vitro research can offer a clearer understanding of the direct effects of TXA on infection rates. Such studies are crucial for determining the causative relationships and the underlying mechanisms by which TXA might influence patient outcomes. This understanding is pivotal in developing targeted interventions that can effectively reduce the risk of infection while optimizing the therapeutic use of TXA in hip arthroplasty.

On the same note, an intriguing aspect of TXA’s potential in reducing postoperative complications is its impact on biofilm formation. Biofilm formation is a common cause of chronic PJI and implant failures. Recent studies suggest that TXA may exert an inhibitory effect on biofilm formation, a crucial step in the pathogenesis of PJIs. This effect is thought to be mediated by the reduction of protein and polysaccharide contents within the biofilms, as demonstrated in both in vitro and in vivo models [23]. The ability of TXA to potentially disrupt biofilm integrity could elucidate the observed reduction in postoperative infection rates, highlighting a dual role for TXA in surgical settings: minimizing perioperative blood loss and acting against a critical factor in infection development.

In hip arthroplasty, the utilization of TXA represents a multifaceted cost/benefit profile that extends beyond its primary hemostatic effect. The direct cost savings from reduced intraoperative blood loss and minimized need for postoperative transfusions are clear, but the benefits of TXA use permeate deeper into the patient care continuum. By potentially lowering the incidence of transfusion-related complications, TXA indirectly reduces the risk of immunomodulation and subsequent infection rates, which in turn can lead to shorter hospital stays and fewer readmissions. Furthermore, the reduced occurrence of SSIs mitigates the substantial financial burden associated with long-term antibiotic therapy, additional surgeries, and extended rehabilitation. Thus, the judicious application of TXA in hip arthroplasty encompasses a holistic approach to patient care, promising both clinical efficacy and economic efficiency [24].

Despite the consistent association between TXA use and reduced PJI rates, this systematic review has several limitations. The two non-randomized studies had a moderate risk of bias, with MINORS scores ranging from 10 to 14, primarily due to their retrospective designs, lack of prospective data collection, and potential for selection bias. The only RCT included had a low overall risk of bias but raised concerns regarding randomization and allocation concealment. The absence of PJI cases in this trial further limits its ability to definitively assess TXA’s effect on infection rates, and its small sample size may not be representative of broader clinical practice. Additionally, heterogeneity in TXA administration routes and dosages complicates interpretation, as intravenous and topical TXA were the most commonly evaluated, while oral TXA remains less studied in infection prevention. Variability in follow-up durations across studies, ranging from 30 to 90 days or longer, further impacts comparability and the ability to determine the long-term effects of TXA on infection risk.

A major limitation of this systematic review is the limited number of studies included, which restricts the strength of our conclusions regarding TXA’s effect on infection rates in THA. This was primarily due to the lack of separate reporting for THA and TKA outcomes in many studies. A significant number of otherwise relevant articles (4 studies) had to be excluded because they combined TKA and THA data without stratification, preventing the extraction of THA-specific infection rates [25,26,27,28]. This limitation underscores a broader issue in the literature, where arthroplasty outcomes are often analyzed collectively despite differences in surgical approach, perioperative factors, and infection risks between hip and knee arthroplasty. Future studies should aim to report infection outcomes separately for THA and TKA to allow for more precise assessments of TXA’s impact in each procedure.

The current landscape of literature on the efficacy of TXA in THA is marked by a paucity of high-caliber studies, particularly randomized controlled trials (RCTs), which are considered the gold standard of evidence-based medicine. This deficiency is largely attributable to the entrenched position of TXA in clinical practice, where its benefits in reducing perioperative blood loss are well-recognized, thereby rendering the withholding of TXA for a control group in RCTs ethically contentious. Ethical mandates require clinicians to provide the best-known care to patients, which includes the use of TXA, based on substantial evidence of its efficacy. Consequently, the execution of prospective RCTs is complicated by these ethical obligations, as placing patients in a control group might be considered a denial of standard care. Despite these, we believe that there is a place for RCTs’ when it comes to analyzing dosage and route of administration of TXA and observing the outcomes.

## 5. Conclusions

In conclusion, this systematic review suggests that tranexamic acid may have a protective effect against postoperative infection rates following THA. Future high-quality RCTs are needed to confirm these results and establish optimal dosing and administration protocols.

## Figures and Tables

**Table 1:** Inclusion and exclusion criteria for our systematic review.

Criteria	Inclusion Criteria	Exclusion Criteria
Language	English	Non-English
Subjects	Human	Non-human
Level of Evidence	Levels I-IV, full text available	Level V, no full text available
Procedure	Primary THA	Revision THA for any reason, THA for hip fractures, associated conditions (local bone tumors, coagulation pathology, post-traumatic deformity)
Intervention	TXA	no TXA
Outcomes	SSI or PJI or other type of infections	SSI or PJI or other type of infections not reported separately

**Table 2:** Synthesis of the results from the included manuscripts.

Author and Year		Hsu 2024 [15]	Thepalyia 2024 [16]	Luo 2018 [17]
Type of study		Retrospective cohort	Retrospective cohort	RCT
Level of evidence		III	III	I
All patients	Tot	1766	144,344	117
	Age (yr)	n/a	63.4 ± 11.6 *	n/s
	Sex M/F *	n/s	46/52 **	n/s
Route of administration (comparison)		IV TXA vs. topical vs. no TXA	TXA (n/s) vs. no TXA	Oral TXA vs. topical TXA
Subgroups	Control	797	72,172	n/a
	Intervention	969	72,172	117
	IV TXA	777	n/s	n/a
	Oral TXA	n/a	n/s	59
	Topical TXA	192	n/s	58

Data presented as * mean ± standard deviation (min-max); ** data expressed as %; Abbreviations: IV, intravenous; n/a, not applicable; n/s, not specified; RCT, randomized controlled trial; TXA, tranexamic acid.

**Table 3:** Synthesization of the results from the included manuscripts.

Main Author		Hsu 2024 [15]	Thepalyia 2024 [16]			Luo 2018 [17]
Type of study		Retrospective cohort	Retrospective cohort			RCT
Tot Nr.		1766	144,344			117
Duration of Observation		>12 months	30 & 90 days			90 days
TXA Dosage	IV	10 mg/kg, 10 min before skin incision	n/s			n/a
	Oral	n/a	n/s			2 g 2 h preoperatively, and two doses of 1 g postoperatively
	Topical	1.5–3 g injected intra-articularly or into the drainage tube during surgery	n/s			3 g in the operating room
SSI	No TXA	n/a	n/a			n/a
	TXA		Superficial SSI			
			0.1% *p* < 0.001 * (30 days)			
			0.3%, *p* < 0.001 * (90 days)			
			Deep SSI			
			0.1% *p* = 0.228 (30 days)			
			0.1%, *p* = 0.012 * (90 days)			
	IV TXA		n/a			
	Oral TXA		n/a			
	Topical TXA		n/a			
PJI	No TXA	1.3%				n/a
	TXA	0.6%; *p* value = 0.209	0.6%, *p* = 0.001 * (30 days)			
			1.2%, *p* = 0.019 * (90 days)			
	IV TXA	0.8%; *p* value = 0.426	n/a			
	Topical TXA	0%; *p* value = 0.995 **	n/a			
	IV TXA—Topical TXA	*p* value = 0.995 **	n/a			
Postoperative Infection	Oral TXA	n/a	n/a	n/a	n/a	0
	Topical TXA					0
Conclusion		**Favors TXA for PJI** *but not statical significance*	**Favors TXA for superficial SSI**	**Favors TXA for deep SSI**	**Favors TXA for PJI**	*No difference between oral* vs. *topical TXA*

* Statistical significance; ** The model failed because of the small sample size; Abbreviations: IV, intravenous; n/a, not applicable; n/s, not specified; PJI, periprosthetic joint infection; RCT, randomized controlled trial; SSI, surgical site infection; TXA, tranexamic acid.

**Table 4:** MINORS assessment of our non-randomized studies manuscripts.

Main Author/MINORS Item	Hsu 2024 [15]	Thapaliya 2024 [16]
Clearly stated aim	2	2
Inclusion of consecutive patients	0	2
Prospective data collection	2	0
Endpoints appropriate to the aim	2	2
Unbiased assessment of endpoints	0	2
Appropriate follow-up period	2	2
Loss to follow-up < 5%	0	2
Prospective calculation of study size	2	2
**Total Score** (out of 16)	**10**	**14**

## Supplementary Materials

The following supporting information can be downloaded at: https://www.mdpi.com/article/10.3390/jcm14092910/s1. File S1: Search strategy, File S2: Search screening.
