# Supplementary material for: The Influence of Tranexamic Acid (TXA) on Postoperative Infection Rates Following Total Hip Arthroplasty (THA)—A Systematic Review
Source: J Clin Med. 2025 Apr 23;14(9):2910. doi: 10.3390/jcm14092910 (PMC12072976; doi:10.3390/jcm14092910)
Supplement: Supplementary file 1 [file jcm-14-02910-s001.zip › jcm-3571177-supplementary/SF1 - Search strategy.pdf]

Searches
From inception up until December 25, 2024

PubMed
("Tranexamic Acid"[Mesh] OR "Tranexamic Acid"[tiab] OR TXA[tiab]) AND ("Periprosthetic Joint Infection"[tiab] OR PJI[tiab] OR "Surgical Wound Infection"[Mesh] OR "Postoperative Wound Infection*"[tiab] OR "Wound Infection*"[tiab] OR "Surgical Site Infection*"[tiab] OR "Joints"[Mesh] OR "Hip Joint"[Mesh]) AND ("Arthroplasty, Replacement, Hip"[Mesh] OR "total hip arthroplast*"[tiab] OR "Total Hip Replacement*"[tiab] OR "Primary Hip Arthroplasty"[tiab])
37 results

Scopus
(TITLE-ABS-KEY (tranexamic AND acid) AND TITLE-ABS-KEY (total AND hip AND arthroplasty) AND TITLE-ABS-KEY (periprosthetic AND joint AND infection)) AND (LIMIT-TO (DOCTYPE , "ar") OR LIMIT-TO (DOCTYPE , "re")) AND (LIMIT-TO (LANGUAGE , "English")) AND (LIMIT-TO (EXACTKEYWORD , "Tranexamic Acid"))
85 results

Web of Science
(((ALL=(tranexamic acid OR TXA)) AND (ALL=(total hip replacement OR total hip arthroplasty)) AND ((ALL=(periprosthetic joint infection OR surgical site infection OR wound infection)))
64 results

Cochrane
("tranexamic acid" OR "TXA") AND ("total hip arthroplasty" OR "total hip replacement") AND ("postoperative infection" OR "surgical site infection" OR "wound infection" OR "periprosthetic joint infection") in Title Abstract Keyword
8 results

Epistemonikos
(title:(tranexamic acid) OR abstract:(tranexamic acid)) AND (title:(total hip arthroplasty) OR abstract:(total hip arthroplasty)) OR (title:(total hip replacement) OR abstract:(total hip replacement)) AND (title:(surgical site infection) OR abstract:(surgical site infection)) OR (title:(postoperative infection) OR abstract:(postoperative infection)) OR (title:(periprosthetic joint infection) OR abstract:(periprosthetic joint infection)) OR (title:(wound infection) OR abstract:(wound infection))
83 results
